# Assessment of Wound-Healing Properties of Medicinal Plants: The Case of *Phyllanthus muellerianus*

**DOI:** 10.3389/fphar.2018.00945

**Published:** 2018-08-21

**Authors:** Yaw D. Boakye, Christian Agyare, George P. Ayande, Nicholas Titiloye, Emmanuel A. Asiamah, Kwabena O. Danquah

**Affiliations:** ^1^Department of Pharmaceutics, Kwame Nkrumah University of Science and Technology, Kumasi, Ghana; ^2^School of Pharmacy, University of Nottingham, Nottingham, United Kingdom; ^3^Department of Pathology, Kwame Nkrumah University of Science and Technology, Kumasi, Ghana; ^4^School of Allied Health Sciences, University of Health and Allied Sciences, Ho, Ghana; ^5^Department of Medical Laboratory, Kwame Nkrumah University of Science and Technology, Kumasi, Ghana

**Keywords:** *Phyllanthus muellerianus*, geraniin, TGF-β_1_, collagen, hydroxyproline, excision model, incision model, cytotoxicity

## Abstract

*Phyllanthus muellerianus* (Family Euphorbiaceae) is a shrub, which is widely distributed in West Africa and employed traditionally as a wound-healing agent especially in Ghana. The aim of the study was to determine the *in vivo* wound-healing activity of aqueous aerial part extract of *P. muellerianus* (PLE) and its major isolate, geraniin. Excision and incision wound models were used to determine the wound-healing activity. Wounds were treated with PLE (0.25, 0.5, and 1% w/w) and geraniin (0.1, 0.2, and 0.4% w/w) aqueous creams. PLE and geraniin significantly (*p* < 0.001) increased wound contraction rate and hydroxyproline production compared to untreated wounds. Histological studies of wound tissues showed high levels of fibroblasts and increased collagen content and cross-linking in PLE and geraniin-treated wound tissues. Immuno-histochemical investigations revealed high levels of TGF-β_1_ in PLE and geraniin-treated wound tissues compared to the untreated wound tissues. Tensile strength of incised wounds was significantly (*p* < 0.05) high in PLE and geraniin-treated wounds. PLE (0.1–100 μg/mL) significantly (*p* < 0.001) reduce LDH release from HaCaT-keratinocytes compared to the untreated cells. PLE and geraniin possess wound healing and cytoprotective effect.

## Introduction

Wounds represent a major global health challenge, which put much economic, financial, and social stress on health institutions, care-givers, patients, and their families (Benbow, 2011). Wounds are defined as physical, chemical, or thermal injuries or insult that result in an opening or breaking in the integrity of the skin or the disruption of anatomical and functional integrity of living tissues (Meenakshi et al., 2006).

The use of medicinal plants in the management of acute and chronic wounds is common in most traditional medicine practices in the world. Based on this, many plants in the tropical and subtropical regions of the world have been screened for their wound-healing activity (Agyare et al., 2009; Farzaei et al., 2014). There are a lot of medicinal plants to be screened in the search for newer, efficacious, and cost effective wound-healing agents.

*Phyllanthus muellerianus* (Kuntze) Exell, which belongs to the family Euphorbiaceae, is widely distributed in West Africa. It is used traditionally in treating wounds in Ghana and other parts of West Africa (Agyare et al., 2009). It is also used to manage menstrual disturbances, pain, dysentery, gonorhoea, and stomach sores. Freshly ground leaves are applied to boils, wounds, and also used for treatment of menstrual disorders, fevers, and skin eruptions in Sierra Leone, Ghana, Nigeria, and Cameroon (Burkill, 2000; Agyare et al., 2009). Fresh leaves and decoction are used to treat constipation, bronchitis, and urethral discharges (Chopra et al., 1986; Siram et al., 2004). The bark of *P. muellerianus* has been shown to contain 22β-hydroxyfriedel-1-ene, 1β, 22β-hydroxyfriedel-1-ene (Adesida et al., 1972). In addition, geraniin, furosin, corilagin, isoquercitrin, astragalin, rutin, phaselic acid, gallic acid, methylgallate, caffeic acid, chlorogenic acid, 3,5-o-dicaffeoylquinic acid have been isolated from the leaves and aerial parts of *P. muellerianus* (Agyare et al., 2011). Saleem et al. (2009) also isolated bis (2-ethyloctyl) phthalate, bis (2-ethylicosyl) phthalate, 3-friedelanone, methylgallate, β-sitosterol from the leaves of *P. muellerianus*.

Agyare et al. (2011) reported that the aqueous leaf extract of *P. muellerianus* and its major isolate, geraniin stimulate cellular activity, differentiation, and collagen synthesis of human skin keratinocytes and dermal fibroblasts. The anti-infective (Boakye et al., 2016c), anti-inflammatory (Boakye et al., 2016a), and antioxidant activities (Wang et al., 2015; Boakye et al., 2016b) of *P. muellerianus* and geraniin have also been reported. However, the folkloric use of *P. muellerianus* as a wound-healing agent has not been scientifically proven in *in vivo* models. Hence, the aim of the study was to investigate the *in vivo* wound-healing activity of *P. muellerianus* and its major metabolite, geraniin.

## Materials and Methods

### Plant Collection

The fresh matured aerial parts of *P. muellerianus* were collected from the Kuntunase community (longitude 1.0° 28′18″W and latitude 6.0° 32′23″N), Ashanti Region, Ghana in February 2010. The plant was authenticated and a voucher specimen (A 001) deposited at Ghana Herbarium, University of Ghana, Accra, Ghana.

### Preparation of Aqueous Extract

The plant material was washed under running water, air-dried at room temperature and ground to powder (1 kg) using a lab mill machine (Christy and Norris, United Kingdom). The aqueous extract of the plant was prepared according to method described by Agyare et al. (2011). Powdered plant material was extracted in 10 L of distilled water by heating at 90°C for 15 min under atmospheric pressure. The extract was filtered using a Buchner funnel and Whatman no. 10 filter paper and concentrated under reduced pressure at 45°C by a vacuum rotary evaporator (R-210, BUCHI, Switzerland) and further lyophilized using a freeze drying system to yield the powdered plant extract (14.1% w/w), which will subsequently be referred as PLE.

### Source of Geraniin

The geraniin (96% w/w HPLC grade) used in this study was kindly provided by Prof. Andreas Hensel, Institute of Pharmaceutical Biology and Phytochemistry, University of Muenster, Muenster, Germany. Geraniin was isolated from the aqueous extract of the aerial parts of *P. muellerianus* (Kuntze) Exell as describe by Agyare et al. (2011).

### Experimental Animals

Male Sprague-Dawley rats (200–250 g) were acquired from Noguchi Memorial Institute for Medical Research, University of Ghana, Legon, Accra, Ghana. The rats were kept in groups of five in clean polypropylene cages in the experimental animal house of the Department of Pharmacology, College of Health Sciences, Kwame Nkrumah University of Science and Technology, Kumasi, Ghana under temperature of 25°C, normal daylight, and relative humidity of 55–60%. Rats were fed on commercial pellets (GAFCO, Ghana) *ad libitum*, with clean water readily accessible to the caged rats in clean bottles. Animals were allowed to acclimatize for a week before experiment was performed.

### Ethical Approval

The animal studies were approved by Animal Ethical Committee (FPPS-AEC/CA01/13), Faculty of Pharmacy and Pharmaceutical Sciences, Kwame Nkrumah University of Science and Technology, Kumasi, Ghana. All experimental protocols were performed and observed according to internationally accepted principles for laboratory animal use and care (EEC Directive of 1986: 86/609 EEC). All animals were euthanized at the end of the experiment.

### Excision Wound Model

The excision wound model as described by Morton and Malone (1972) and Agyare et al. (2013) was employed in this study. The dorsal fur of the rats was trimmed and depilated using clean razor blades. The shaved area was then treated with 70% v/v ethanol (Merck BDH, Poole, United Kingdom). The rats were anaesthetized with an intramuscular injection of ketamine hydrochloride (Pfizer, New York, NY, United States) at a dose of 50 mg/kg body weight. With the aid of a pair of sterile surgical scissors and toothed forceps, full thickness wounds of approximately 20 mm in diameter were excised at the back of each rat to a depth of loose subcutaneous tissue. Hemostasis was achieved by even compression with sterile gauze and wounds were left untreated for a period of 24 h. Rats were randomized into 9 groups consisting of 10 rats per group and topically treated with aqueous cream base (vehicle), silver sulphadiazine (1% w/w) cream (Ayrton drugs, Accra, Ghana), PLE (0.25, 0.50, and 1.0% w/w) aqueous creams, geraniin (0. 1, 0.2, and 0.4% w/w) creams and untreated group (control). All the wounds received daily standard wound cleansing with 0.9% w/v saline solution (intravenous infusions, Koforidua, Ghana) prior to the topical treatment with the aqueous creams (0.1 g of aqueous cream per daily treatment). Any rat showing a wound hematoma or wound infection was immediately euthanized with an overdose of ketamine hydrochloride (200 mg/kg) to avoid any discomfort. Also, data from these animals were not used in the final analysis. Wound scar tissues were cut on the 13th day post wounding for histological studies and hydroxyproline content. For evaluation of beta-1 transforming growth factor (TGF-β_1_), wound scar tissues were harvested on the 7th day post-injury, which is the approximate timing of the peak of TGF-β_1_ expression and half timing of complete wound closure. Harvested wound tissues from the various groups were stored immediately at -20°C till laboratory analysis.

#### Assessment of Wound Diameter

The diameters of the excised wounds were measured with the aid of a millimeter rule on days 1, 3, 5, 7, 9, 11, and 13 post-injury and photographed using a Kodak Digital Science DC260 Zoom camera (Kodak, Rochester, NY, United States).

#### Determination of Hydroxyproline Content

The amount of hydroxyproline in both treated and untreated wound tissues was determined according to the method described by Woessner (1961) and modified by Gurung and Skalko-Basnet (2009). Wound tissues were excised on day 13 post-injury and stored at -20°C till laboratory analysis. Samples were dried in a hot air oven at 60–70°C to constant weight and were hydrolyzed in 6 M HCl (Sigma-Aldrich, Michigan, United States) at 130°C for 3 h in sealed test tubes. The hydrolysate was neutralized to pH 7 with 2.5 M NaOH (Sigma-Aldrich, London, United Kingdom). Two milliliters of sample was transferred into a test tube and subjected to chloramine-T (Sigma-Aldrich, Michigan, United States) oxidation for 20 min at 25°C. The reaction was terminated by addition of 1 mL of 3.15 M per chloric acid (Fisher Scientific, Glasgow, United Kingdom) and allowed to stand for 5 min at 25°C. One milliliter of Ehrlich reagent (Sigma-Aldrich, St. Louis, Missouri, United States) was added, shaken, and incubated at 60°C for 20 min. The pink color developed after incubation was measured at 557 nm using a LAMBDA 1050 UV-visible spectrophotometer (PerkinElmer, 940 Winter St. Waltham, Massachusetts, United States). The amount of hydroxyproline present in the tissue sample(s) was calculated from a standard curve prepared from pure L-hydroxyproline (Sigma-Aldrich, Michigan, United States).

#### Histology Studies

Wound tissue samples were cut on the 13th day post wounding from all the groups and immediately fixed in 10% buffered formalin (Sigma-Aldrich, Michigan, United States). The fixed tissues were dehydrated in increasing concentration grades of ethanol (Merck BDH, Poole, United Kingdom). The tissues were later embedded in paraffin wax allowed to solidify and cut to 5 μm sections with a Leica rotary microtome (Boston Lab. Equipment, Boston, United States). The tissues were then mounted on a clean glass slide after removing the paraffin and stained with hematoxylin and eosin (Sigma-Aldrich, St. Louis, Missouri, United States). The glass slides were then observed under the light microscope (Leica Microsystems, Wetzlar, Germany). The tissues were observed for the degree of cell repair; re-epithelialization, angiogenesis, collagen content, and granular tissue formation. The procedure was repeated using Verhoeff Van Gieson’s stain (Sigma-Aldrich, St. Louis, MO, United States) to evaluate level of collagen fiber biosynthesis, deposition, and organization (Udupa et al., 1995; Talekar et al., 2012).

#### Immunohistochemistry

To evaluate the TGF-β_1_ levels on wound tissues, avidin biotin complex (ABC) immunochromogenic staining was performed according to the method described by Bratthauer (2010) using human anti mouse monoclonal antibody for TGF-β1 (R&D Systems, Minneapolis, MN, United States). Briefly, tissue sections were incubated with the primary human anti mouse monoclonal antibody TGF-β1 (1:100 dilution, R&D Systems, Minneapolis, MN, United States) overnight at 4°C, followed by incubation with biotinylated secondary antibody at 25°C for 1 h. The slides were then incubated with streptavidin–horseradish peroxidase (HRP) at 25°C for 15 min, washed three times in phosphate buffered saline (PBS), and incubated with substrate chromogen solution 3, 3-diaminobenzidine (DAB) at 25°C for 5 min and counterstained with Mayer hematoxylin (Sigma-Aldrich, St. Louis, Missouri, United States) for 3 min. The slides were then viewed with the aid of an Axioscop Zeiss microscope (Carl Zeiss Microscopy, Thornwood, United States), and TGF-β_1_ levels in wound tissues were assessed blindly by a histologist. Representative images of the sectioned wound tissues were captured using the software Axiovision (Carl Zeiss Microscopy, Thornwood, United States).

### Incision Wound Model

The incision wound-healing model as described by Ehrlich and Hunt (1968) was employed in this study. The dorsal fur of the rats was depilated using clean razor blades; the shaved area was then treated with 70% ethanol (Merck BDH, Poole, United Kingdom). The rats were anaesthetized with an intramuscular dose of 50 mg/kg body weight ketamine hydrochloride (Pfizer, New York, United States). With the aid of surgical blade, 6 cm long paravertebral incisions were made through the full thickness of the skin on each side of the vertebral column. After incision, the parted skin was surgically sutured 1 cm apart using a surgical thread and curved needle. Rats were randomized into nine groups consisting of five rats per group. The incised wounds were subsequently topically treated with aqueous cream base (vehicle), silver sulphadiazine (1% w/w) cream, PLE (0.25, 0.50, and 1.0% w/w) aqueous creams, geraniin (0.1, 0.2, and 0.4% w/w) aqueous creams, and untreated group (control). The sutures were removed on the 7th day post wounding. Wound tensile strength was measured on the day 10 post wounding. Any rat showing a wound hematoma or wound infection was immediately euthanized with an overdose of ketamine hydrochloride to avoid any discomfort. Also, data from these animals were not used to assess wound healing.

#### Tensile Strength

The tensile strength was determined using the method described by Lee (1968) with slight modification. Fine sand of particle size 0.25 mm was used in place of water to determine the tensile strength of the incised wounds. Rats were anesthetized with ketamine hydrochloride (50 mg/kg) and secured to the operation table with the aid of clippers. Two forceps were firmly applied, 2 mm away from each side of the incised wound area. One of the forceps was fixed whiles the other forceps was connected to a freely suspended light weight polypropylene container with the aid of a 100-cm string. Fine sand was allowed to flow from the reservoir slowly and steadily into the polypropylene container until the wound just opened up.

### Influence of PLE and Geraniin on HaCaT-Keratinocytes

HaCaT-keratinocytes were seeded in 96-well tissue culture plates at a concentration of 5000 cells per 100 μL HaCaT-keratinocytes growth medium (PAA Laboratories Pasching, Austria) and then incubated in 5% CO_2_ at 35°C for 48 h. The cells were then washed with 100 μL PBS. The cells were fed with 100 μL test solution/medium at different concentrations of extracts and compound and then further incubated for 72 h. The culture medium was used as untreated control and 1% fetal calf serum (FCS) in test medium used as a positive control.

#### Cytotoxicity Studies

This test was performed using HaCaT-keratinocytes (6 × 10^3^ cells/mL) as described by Decker and Lohmann-Matthes (1988) using cytotoxicity detection (LDH) kit (Roche Diagnostics GmbH, Mannheim, Germany). The cells were cultured in 96-well micro-titer plates (MTP) containing graded concentration of test compound and incubated at 35°C for 24 h. After incubation, 25 μL of each test medium (supernatant) was transferred into a 96-well MTP. To the remaining medium, 25 μL cell lysis solution (10% Triton X-100 in 5% FCS) was added. This was incubated for 90 min at room temperature with constant shaking. Twenty-five microliters reaction mixture were added to both the supernatant and lysed cell medium and incubated in the dark at 20°C for 30 min. The reaction was terminated by the addition of 10 μL 1 M HCL (Sigma-Aldrich, London, United Kingdom) solution to each well. Precautions were taken to prevent the formation of air bubbles by the addition of 5 μL 96% ethanol to the plates. The absorbance of the resultant solutions was measured with MTP reader at 490 against 690 nm.

### Statistical Analysis

Data were analyzed with Graph Pad Prism Version 5.0 for Windows (Graph Pad Software Inc., San Diego, CA, United States) statistical package program. One-way ANOVA followed by Dunnett’s *post hoc* test was employed in the analysis of data. However, two-way ANOVA followed by Bonferroni’s *post hoc* test was used to analyze the time-course curve for the wound-healing process.

## Results

### Influence of PLE and Geraniin on Rate of Wound Closure

**FIGURE 1:**
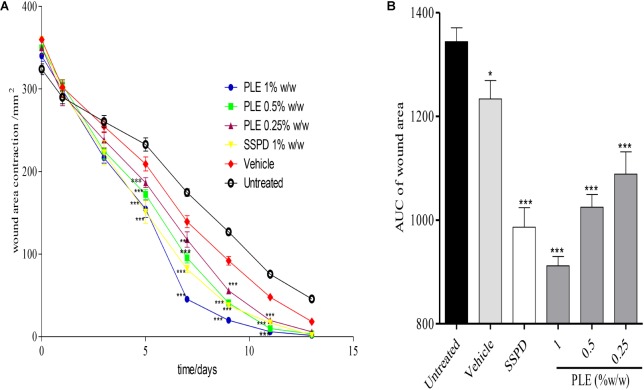
Influence of PLE on rate of wound closure. Full thickness wounds of approximately 20 mm in diameter were excised on the dorsal region of rats. The excised wounds were topically treated with 0.25, 0.5, and 1% w/w PLE formulated as aqueous cream. Wound diameter was measured on days 1, 3, 5, 7, 9, 11, and 13 post injuries. **(A)** Time-course curve, **(B)** area under curve (AUC) of time course curve. PLE: aqueous extract of the aerial parts of *P. muellerianus*. Vehicle group was treated with aqueous cream base. SSPD: 1% w/w silver sulphadiazine-treated wounds (positive control). Values are mean ± SEM, *n* = 5. ^∗∗∗^*p* < 0.001; ^∗∗^*p* < 0.01; ^∗^*p* < 0.05 compared to untreated group (two-way ANOVA followed by Bonferroni’s *post hoc* test).

Wounds treated with 0.25, 0.5, and 1% w/w PLE significantly (*p* < 0.001) reduced wound area from day 5 to 11 post-injury compared to the untreated wounds (**Figure 1A**). Furthermore, the area under the curve (AUC) revealed that 0.25, 0.5, and 1% w/w PLE significantly (*p* < 0.001) reduced wound area compared to the untreated wounds (**Figure 1B**).

**FIGURE 2:**
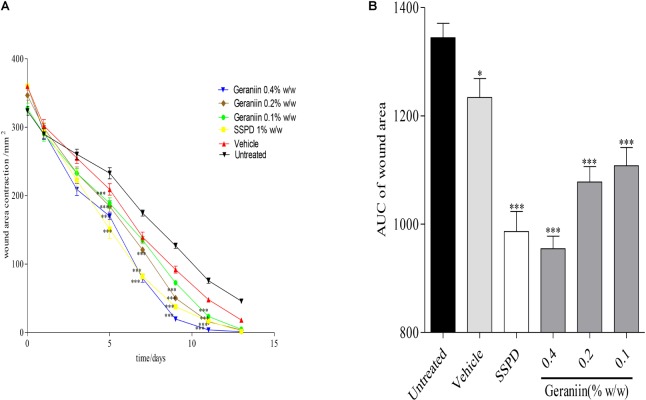
Influence of geraniin on rate of wound closure. Full thickness wounds of approximately 20 mm in diameter were excised on the dorsal region of rats. The excised wounds were topically treated with geraniin (0.1, 0.2, and 0.4% w/w) formulated as aqueous cream. Wound diameter was measured on days 1, 3, 5, 7, 9, 11, and 13 post injuries. **(A)** Time-course curve, **(B)** AUC of time course curve. Excised wounds were treated with 0.1, 0.2, and 0.4% w/w geraniin. Vehicle group was treated with aqueous cream base. SSPD: 1% w/w silver sulphadiazine-treated wounds. Values are mean ± SEM, *n* = 5. ^∗∗∗^*p* < 0.001; ^∗∗^*p* < 0.01; ^∗^*p* < 0.05 compared to untreated group (two-way ANOVA followed by Bonferroni’s *post hoc* test).

In the geraniin-treated group, geraniin at concentrations of 0.1, 0.2, and 0.4% w/w significantly (*p* < 0.001) reduced wound area on days 5, 7, 9, and 11 post-injury compared to the untreated group (**Figure 2A**). Also, the AUC revealed a significant reduction in wound area of 0.1, 0.2, and 0.4% w/w geraniin-treated groups compared to the untreated group (**Figure 2B**).

### Histological Evaluation of Wound Tissues Treated With PLE and Geraniin

**FIGURE 3:**
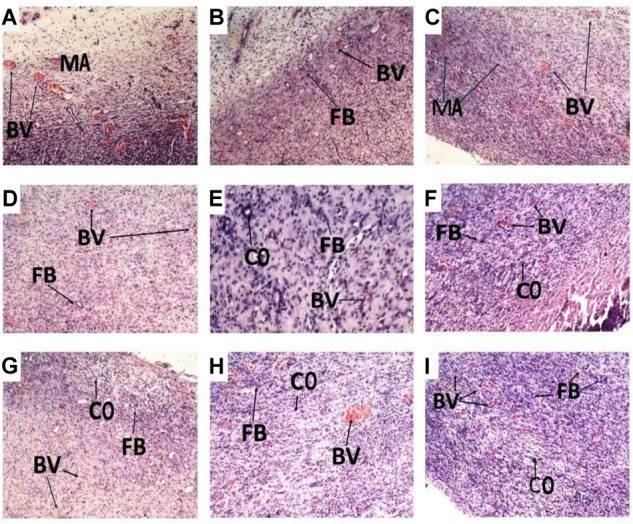
Histological images (×400) representing the influence of PLE and geraniin in excised wound tissues from both treated and untreated rat wounds. Full thickness wounds were created on the dorsal region and topically treated with PLE, geraniin, silver sulphadiazine, vehicle (cream only). Wound tissues were excised 13 days post wounding and sectioned. Serial sections were stained with hematoxylin and eosin and evaluated microscopically for fibroblast proliferation, neovascularization, epithelial regeneration, and collagen deposition (H&E stain ×400). **(A)** Untreated wound tissues, **(B)** vehicle-treated (aqueous cream only) wound tissues, **(C)** 1% w/w silver sulphadiazine-treated wound tissues, **(D)** 0.25% w/w PLE-treated wound tissues, **(E)** 0.5% w/w PLE-treated wound tissues, **(F)** 1% w/w PLE-treated wound tissues, **(G)** 0.1% w/w geraniin-treated wounds tissues, **(H)** 0.2% w/w geraniin-treated wound tissues, and **(I)** 0.4% w/w geraniin-treated wound tissues. CO, collagen strands; MA, macrophages and monocytes; BV, blood vessels; FB, fibroblast.

Histology of excised wound tissues showed improved wound-healing activity (fibroblasts proliferation, neovascularization, epithelial regeneration, and collagen deposition) in PLE and geraniin-treated wounds compared to the untreated group (**Figure 3**).

In the untreated wound tissues, persistent inflammation with marked tissue necrosis and little proliferation at edges of wound, which indicated poor wound-healing rate, was observed (**Figure 3A**). Vehicle-treated wound tissues (aqueous cream only) showed persistent inflammation and little proliferation at wound edges, which indicated poor wound-healing rate (**Figure 3B**). In silver sulphadiazine-treated wound tissues (1% w/w), there were granulation tissue formation and angiogenesis with evidence of fibroblast proliferation and collagen synthesis indicative of marked re-epithelialization at wound edges but not much re-epithelialization in center of wounds (**Figure 3C**).

PLE-treated wound tissues (0.25% w/w) showed appreciable angiogenesis and fibroblastic activity with evidence of substantial collagen deposition in wounds (**Figure 3D**). PLE-treated wound tissues (0.5% w/w) exhibited increased collagenation and re-epithelialization, indicative of high rate of wound healing (**Figure 3E**). In the 1% w/w PLE-treated wound tissues, increase in collagenation and re-epithelialization were observed, which is indicative of high wound-healing rate (**Figure 3F**).

Geraniin-treated wound tissues (0.1% w/w) exhibited appreciable angiogenesis and granulation tissue formation with evidence of high collagen deposition and fibroblastic activity (**Figure 3G**). In the 0.2% w/w geraniin-treated wound tissues, there was an increased fibroblastic activity, increased collagenation, increased angiogenesis, and granulation tissue formation. These indicated high wound-healing activity (**Figure 3H**). Geraniin-treated wound tissues (0.4% w/w) showed increased fibroblastic activity, increased collagenation, increased angiogenesis, and re-epithelialization. These indicated high wound-healing activity (**Figure 3I**).

#### Influence of PLE and Geraniin on Hydroxyproline Production in Wound Tissues

**FIGURE 4:**
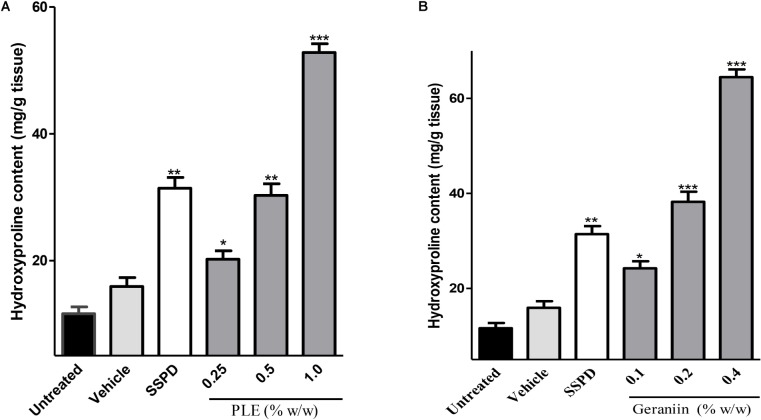
Influence of PLE **(A)** and geraniin **(B)** treatment on hydroxyproline production in excised wound tissues. Hydroxyproline content was determined using acid hydrolysate of excised wound tissue on day 7 post injury. Excised wounds were treated with PLE (0.25, 0.5, and 1% w/w), geraniin (0.1, 0.2, and 0.4% w/w), silver sulphadiazine, and cream only. PLE, aqueous extract of the aerial parts of *P. muellerianus*. Vehicle group was treated with aqueous cream only. SSPD, 1% w/w silver sulphadiazine-treated wounds. Values are mean ± SEM, *n* = 5. ^∗∗∗^*p* < 0.001; ^∗∗^*p* < 0.01; ^∗^*p* < 0.05 compared to untreated wound tissues (One-way ANOVA followed by Dunnet’s *post hoc* test).

There were significant increase in hydroxyproline production in wound tissues treated with 0.25 (*p* < 0.01), 0.5 (*p* < 0.001), and 1% w/w *(p* < 0.001) PLE compared to the untreated wound tissues (**Figure 4A**). Also, 0.1, 0.2, and 0.4% w/w geraniin-treated wound tissues showed significant (*p* < 0.001) increase in hydroxyproline production compared to the untreated wound tissues (**Figure 4B**).

### Influence of PLE and Geraniin on Collagen Production in Wound Tissues

**FIGURE 5:**
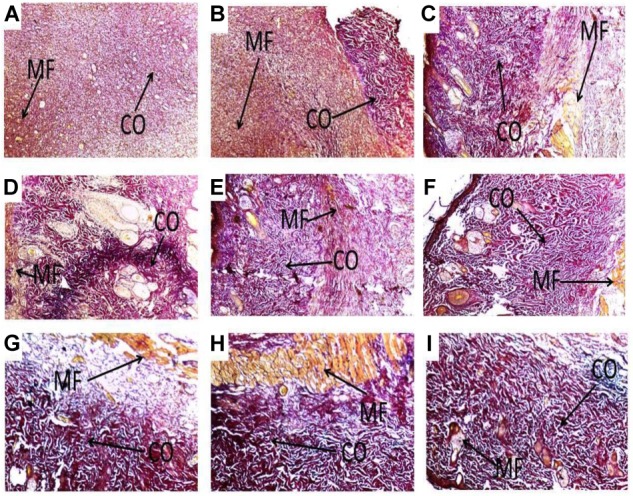
Histological images showing influence of PLE and geraniin on collagen production in both PLE and geraniin-treated and untreated wound tissues. Full thickness wounds were created on the dorsal region and topically treated with PLE, geraniin, silver sulphadiazine, vehicle (cream only). Wound tissues were excised 13 days post wounding and sectioned. Serial sections were stained with Verhoeff–Van Gieson stain and evaluated microscopically for degree collagen deposition (Van Gieson stain ×400). Wound tissues were blindly examined by a pathologist. **(A)** Untreated wound tissues, **(B)** vehicle-treated (aqueous cream only) wound tissues, **(C)** 1% w/w silver sulphadiazine-treated wound tissues, **(D)** 0.25% w/w PLE-treated wound tissues, **(E)** 0.5% w/w PLE-treated wound tissues, **(F)** 1% w/w PLE-treated wound tissues, **(G)** 0.1% w/w geraniin-treated wound tissues, **(H)** 0.2% w/w geraniin-treated wound tissues, and **(I)** 0.4% w/w geraniin-treated wound tissues. CO, collagen fibers; MF, muscle fibers.

Wounds treated with PLE and geraniin showed increased collagen deposition and crosslinking compared to the untreated wounds (**Figure 5**). The untreated wound tissues exhibited little collagen fibers indicative of poor wound-healing rate (**Figure 5A**). There were localized collagen fibers, which indicated slow wound-healing rate in the vehicle-treated (aqueous cream only) wound tissues (**Figure 5B**). Silver sulphadiazine-treated wound tissues (1% w/w) showed generalized collagen fibers in wound bed indicating high wound-healing rate (**Figure 5C**).

Additionally, PLE-treated wound tissues (0.25% w/w) exhibited high collagen fiber accompanied with observable tight cross links of collagen fibers (**Figure 5D**). High collagen fiber content accompanied with observable tight intercalation of collagen fibers in wound bed indicative of high fibroblastic activity was observed in the PLE-treated wound tissues (0.5% w/w) (**Figure 5E**). PLE-treated wound tissues (1% w/w) exhibited high collagen fiber deposition in wound bed with intercalation. This indicated high wound-healing activity (**Figure 5F**).

Geraniin-treated wound tissues (0.1 and 0.2% w/w) showed high collagen fiber accompanied with tight cross links of collagen fibers (**Figures 5G,H**). Geraniin-treated wound tissues (0.4% w/w) exhibited high collagen fiber deposition in wound bed with intercalation. This indicated high wound-healing activity (**Figure 5I**).

### Influence of PLE and Geraniin Treatment on TGF-β_1_ Levels

**FIGURE 6:**
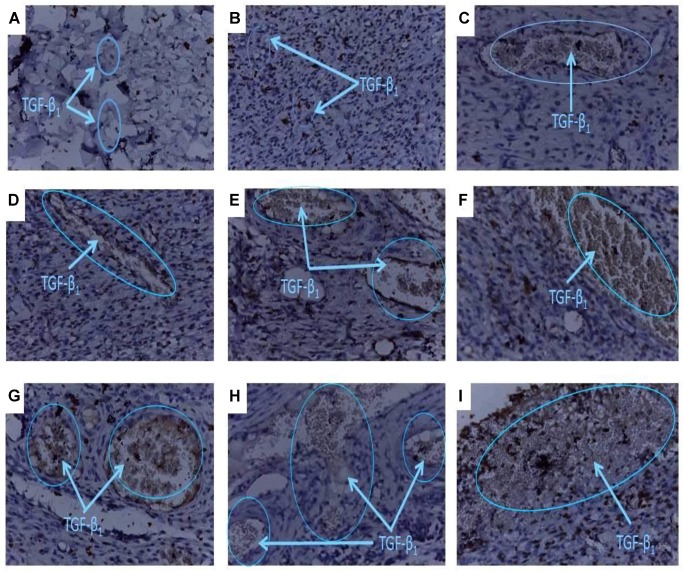
Histology of wound tissues showing influence of PLE and geraniin on TGF-β_1_ levels in excised wound tissues. Full thickness wounds were created on the dorsal region and topically treated with PLE, geraniin, silver sulphadiazine, vehicle (cream only). Wound tissues were excised on day 7 post wounding and sectioned. Serial sections were incubated in the primary antibody overnight. Sectioned tissues were then incubated in the biotinylated secondary antibody, which was further conjugated with HRP to form a complex. The addition of DAB to the complex produced a brownish chromophore and counter stained with Mayers haematoxylin. The slides were blindly examined under the microscope. Region of positive immunoreactivity (brownish chromophore) is indicated by the blue line. Representative images of the treatment groups revealed the following morphological features: **(A)** Untreated-wound tissues, **(B)** vehicle-treated (aqueous cream only) wound tissues, **(C)** 1% w/w silver sulphadiazine-treated wound tissues, **(D)** 0.25% w/w PLE-treated wound tissues, **(E)** 0.5% w/w PLE-treated wound tissues, **(F)** 1% w/w PLE-treated wound tissues, **(G)** 0.1% w/w geraniin-treated wound tissues, **(H) –**0.2% w/w geraniin-treated wound tissues, and **(I)** 0.4% w/w geraniin-treated wound tissues.

Representative images revealed improved TGF-β_1_ levels in wounds treated with PLE and geraniin compared to the control (**Figure 6**). Representative images of the treatment groups revealed the following morphological features.

Untreated wound tissues showed little or no observable TGF-β_1_ immunoreaction in excised wound tissue indicative of very low TGF-β_1_ concentration in wounds (**Figure 6A**). Vehicle-treated (aqueous cream only) wound tissues exhibited little or scanty TGF-β_1_ immunoreaction in excised wound tissue indicative of very low TGF-β_1_ levels in wound (**Figure 6B**). Silver sulphadiazine-treated wound tissues (1% w/w) exhibited localized TGF-β_1_ immunoreaction in wound bed which indicated appreciable TGF-β_1_ levels (**Figure 6C**).

PLE-treated wound tissues (0.25% w/w), however, showed low TGF-β_1_ immunoreactions in wound bed evidence of low TGF-β_1_ levels in wound tissues (**Figure 6D**). PLE-treated wound tissues (0.5% w/w) exhibited localized TGF-β_1_ immunoreactions in wound bed evidence of appreciable TGF-β_1_ levels (**Figure 6E**). PLE-treated wound tissues (1% w/w) also exhibited high TGF-β_1_ immunoreactions in wound bed. This indicated high TGF-β_1_ levels in wound tissues (**Figure 6F**).

Additionally, geraniin-treated wound tissues (0.1% w/w) showed moderate TGF-β_1_ immunoreactions. This indicated appreciable TGF-β_1_ concentration in wound tissues (**Figure 6G**). Geraniin-treated wound tissues (0.2% w/w) exhibited marked TGF-β_1_ immunoreactions. This indicated appreciable TGF β_1_ concentration in wound tissues (**Figure 6H**). Geraniin-treated wound tissues (0.4% w/w) showed profuse TGF-β_1_ immunoreactions indicating high TGF β_1_ levels in wound tissues (**Figure 6I**).

### Influence of PLE and Geraniin on Tensile Strength of Incised Wound Tissues

**FIGURE 7:**
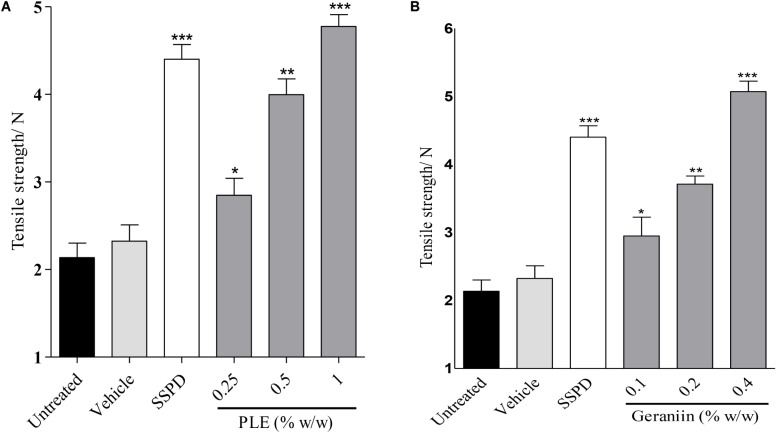
Influence of PLE **(A)** and geraniin **(B)** on tensile strength of incised wound tissue. Paravertebral incisions were made through the full thickness of the skin on each side of the vertebral column and sutured. The sutured wounds were treated with PLE (0.25, 0.5, and 1% w/w) and geraniin (0.1, 0.2, and 0.4% w/w) and tensile strength determined on 10 days post incision. PLE, aqueous extract of the aerial parts of *P. muellerianus*; SSPD, 1% silver sulphadiazine-treated wound tissues. Vehicle group was treated with aqueous cream base only. Values are mean ± SEM, *n* = 5. ^∗∗∗^*p* < 0.001; ^∗∗^*p* < 0.01; ^∗^*p* < 0.05 compared to untreated group (one-way ANOVA followed by Dunnett’s *post hoc* test).

There was significant increase in tensile strength of 0.25 (*p* < 0.05), 0.5 (*p* < 0.01), and 1.0% w/w (*p* < 0.001) PLE-treated wounds compared to the untreated wounds (**Figure 7A**). In addition, there was significant increase in tensile strength in 0.1 (*p* < 0.05), 0.2 (*p* < 0.01), and 0.4% w/w (*p* < 0.001) geraniin-treated wound compared to the untreated wounds (**Figure 7B**).

### Influence of PLE and Geraniin on LDH Release (Cytotoxicity) From HaCaT-Keratinocytes

**FIGURE 8:**
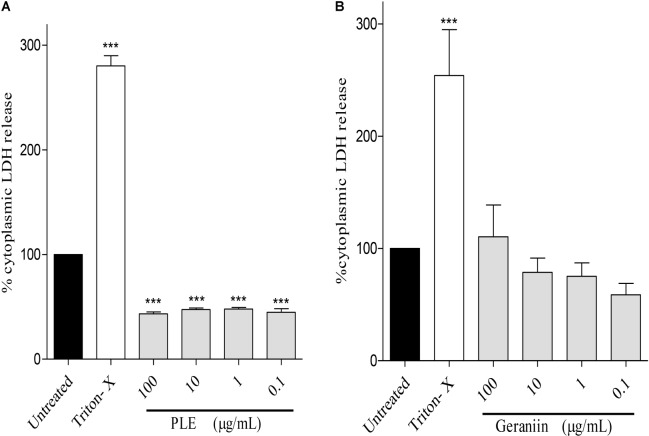
Influence of PLE **(A)** and geraniin **(B)** on LDH release from HaCaT-keratinocytes. HaCaT-keratinocytes were seeded into 96-well plates and incubated to achieve 90% confluency. Cells were then treated with PLE (0.1 to 100 μg/mL) and geraniin (0.1 to 100 μM). Fetal calf serum (1% FCS) was used as positive control. The absorbance was measured at 490 against 690 nm. PLE, aqueous extract of the aerial parts of *P. muellerianus*. Values are mean ± SEM, *n* = 3. ^∗∗∗^*p* < 0.001 compared to untreated cells (one-way ANOVA followed by Dunnett’s *post hoc* test).

PLE significantly (*p* < 0.001) reduced LDH release from HaCaT-keratinocytes at concentrations of 100, 10, 1, and 0.1 μg/mL compared to the untreated cells (**Figure 8A**). However, LDH release from geraniin-treated cells was not significant (*p* > 0.05) compared to the untreated cells (**Figure 8B**).

## Discussion

Wound healing is a complex and dynamic process of restoring tissue structure in damaged tissue as closely as possible to its normal state. It is characterized by hemostasis, inflammation, re-epithelialization, granulation, remodeling of the extracellular matrix, and scar formation (Mary et al., 2002). PLE and geraniin were found to accelerate cutaneous wound healing in rat excision and incision wound models, which are in agreement with *in vitro* stimulatory effect of geraniin and PLE on proliferation of the HaCaT-keratinocytes and primary normal human fibroblasts as reported by Agyare et al. (2011).

Topical application of PLE (0.25, 0.5, and 1% w/w) and geraniin (0.1, 0.2, and 0.4% w/w) aqueous cream formulations on excision wounds in the experimental animals showed significant (*p* < 0.001) reduction in wound area compared to the untreated group (**Figures 1**, **2**), which is an indication of high wound contraction rate. Wound contraction can be defined as the centripetal movement of the edges of a full-thickness wound to facilitate closure of the defect (Suguna et al., 2002; Tang et al., 2007). Wound contraction is therefore an indicator of re-epithelialization, granulation, angiogenesis, fibroblast proliferation, keratinocyte differentiation, and proliferation (Tang et al., 2007). In addition, the pronounced reduction in wound area exhibited by PLE (0.25, 0.5, and 1% w/w) and geraniin-treated (0.1, 0.2, and 0.4% w/w) groups was confirmed by histological studies, which showed high fibroblast proliferation, angiogenesis, and granulation tissue formation, evident of marked re-epithelization, and collagenation in wound bed compared to the untreated group (**Figure 3**).

Fibroblasts stimulate the production or synthesis of collagen, which is a major component of the extracellular matrix. Collagen facilitates the migration of endothelial cells to form new blood vessels to enhance granulation tissue formation and consequently improve wound healing, which is observed as reduction in wound area. However, to estimate the amount of collagen produced in wound bed, hydroxyproline content of wound tissues is always assessed because hydroxyproline is an amino acid, which is almost exclusively confined to collagen (Gao et al., 2006). PLE-treated wound tissues (0.25, 0.5, and 1% w/w) exhibited significant (*p* < 0.05, *p* < 0.01, and *p* < 0.001, respectively) increase in the hydroxyproline production compared to the untreated wound tissues (**Figure 4A**). Also, the geraniin-treated wound tissues (0.1, 0.2, and 0.4% w/w) showed a significant increase in hydroxyproline production (*p* < 0.05, *p* < 0.001, and *p* < 0.001, respectively) compared to the untreated wound tissues (**Figure 4B**). These suggest that both PLE and geraniin were able to influence collagen turnover by increasing collagen biosynthesis, deposition, and maturation and decreasing collagen breakdown by proteases (Gao et al., 2006). The images from the histological sections of the wound tissues suggest that PLE and geraniin accelerated collagen biosynthesis, deposition, and cross-linking compared to the untreated wound tissues (**Figure 5**). Agyare et al. (2011) reported that geraniin stimulates the biosynthesis of collagen from normal human dermal fibroblast, which is in agreement with the findings of this study. Increased collagen deposition and intermolecular crosslinking facilitate the remodeling phase of wound healing since the tensile strength of wounds is attributed to the amount and level of collagen organization or crosslinking in wound bed (Diegelmann, 2001).

Wound-healing processes involve complex interactions between cells as well as various growth factors (Werner and Munz, 2000; Penn et al., 2012). One of the most important factors in the wound-healing process is transforming growth factor-β_1_ (TGF-β_1_) because it is the most abundant isoform in the wound-healing process (Finnson et al., 2013; Ponugoti et al., 2013). The immunohistochemistry (**Figure 6**) of the wound tissues showed an increment in TGF-β_1_ levels in PLE-treated (0.25, 0.5, and 1% w/w) and geraniin-treated (0.1, 0.2, and 0.4% w/w) wound tissues compared to the untreated wound tissues. This may suggest that PLE and geraniin stimulated the expression of TGF-β_1_ in the wound tissues. TGF-β_1_ plays a fundamental role in physiological tissues repair by stimulating fibroblast proliferation, collagen synthesis, production of extracellular matrix, angiogenesis, and wound contraction (Lee et al., 2004; Finnson et al., 2013; Ponugoti et al., 2013). Pastar et al. (2010) and Haroon et al. (2002) reported that chronic, non-healing wounds often show a loss of TGF-β_1_ signaling. In addition, Feinberg et al. (2000) reported that TGF-β_1_ has inhibitory effect on the expression of collagenases and matrix metalloproteinases, which degrade collagen and extracellular matrix. These reports are consistent with the above observations.

Tensile strength, which demonstrates the force per unit of cross-sectional area needed to open closed wounds, is an important measure since it reflects the subdermal organization of the collagen fibers in wound bed (Jimenez and Rampy, 1999). The significant increment of the tensile strength of PLE-treated (0.25, 0.5, and 1% w/w) (*p* < 0.05, *p* < 0.01, and *p* < 0.001, respectively) and geraniin-treated (0.1, 0.2, and 0.4% w/w) (*p* < 0.05, *p* < 0.001, and *p* < 0.001, respectively) wounds compared to the untreated wounds may suggest an improvement in the amount and quality of newly synthesized and deposited collagen (**Figure 7**) (McFarlin et al., 2006). High tensile strength also reflects the extent of intermolecular covalent cross-linking within a fibril and establishment of collagen-ground substance interaction within the wound, such as proteoglycan or glycoprotein (McFarlin et al., 2006). Rashed et al. (2003) also reported that tensile strength is a measure of the quality of the repaired tissue. This may suggest that PLE and geraniin improved the quality of repaired wound tissues.

Wound-healing agents are generally classified as agents that are either able to stimulate fibroblast proliferation, induce keratinocyte cell proliferation and differentiation, increase collagen formation or exhibit antimicrobial, antioxidant and anti-inflammatory activities. The ability of an agent to possess two or more of these biological properties suggest that the agent is potentially a good wound-healing agent (Houghton et al., 2005).

PLE and geraniin have been reported to possess antimicrobial activity against some organisms such as *S. aureus*, *S. pyogenes*, *P. aeruginosa*, *E. coli*, and *C. albicans*, which are implicated in wound contamination and colonization (Boakye et al., 2016c). The presence of these organisms is detrimental to the healing process since these organisms either form biofilms or produce enzymes that destroy the extra cellular matrix (ECM), which subsequently prolong the inflammatory phase and increase oxidative stress in wound bed (Stotts et al., 2007; Guo and DiPietro, 2010). These wounds enter a chronic pathological state and are unlikely to heal without treatment. The antimicrobial activity of PLE and geraniin indicates that PLE and geraniin can be suitable agents that can be used in the management of wounds that are heavily infected with microorganisms.

Again, the reported antioxidant activity of PLE and geraniin may enhance its wound-healing activity. High levels of reactive oxygen species (ROS), such as superoxide anion (O_2_^-^), hydroxyl radicals (OH^-^), singlet oxygen (^1^O_2_), and hydrogen peroxide (H_2_O_2_) in wound site promotes collagen breakdown and hence destruction of the extracellular matrix (ECM). When the ECM is destroyed, processes such as angiogenesis and re-epithelization, which are crucial for wounds to heal are reduced (Siwik et al., 2001; Rodriguez et al., 2008). Boakye et al. (2016b) reported that PLE and geraniin stimulates the production of catalase, superoxide dismutase, and ascorbate peroxidase but reduces the activity of myeloperoxidase and malondialdehyde levels in excised wounds. Geraniin has also been reported to exert cytoprotective effect against cellular oxidative stress by up regulation of Nrf2-mediated antioxidant enzyme expression [heme oxygenase-1 (HO-1), NAD(P)H-dependent quinone oxidoreductase-1 (NQO1), and level of glutathione (GSH)] via PI3K/AKT and ERK1/2 pathway (Wang et al., 2015).

Additionally, in the presence of persistent inflammation, wounds fail to heal and normally enter into a pathological state, which needs treatment to resolve. The ability of an agent to resolve the inflammatory process in wounds will promote or accelerate the healing process (Houghton et al., 2005; Agyare et al., 2013). PLE and geraniin have been reported to exhibit anti-inflammatory activity (Boakye et al., 2016a) and hence may be contributing to their wound-healing activity.

Toxicological evaluations of all medicinal plants and compounds are important in order to ascertain their safety. Low LDH release from HaCaT cells treated with PLE and geraniin (**Figure 8**) is an indication of the ability of the two agents to protect keratinocytes from tissue necrosis, which is characteristic of chronic wounds (Phan et al., 2001; Warriner and Burrell, 2005). This may suggest that PLE and geraniin can be cytoprotective at test concentrations. Agyare et al. (2011) reported that PLE and geraniin increased mitochondrial activity of HaCaT-keratinocytes by increasing cell proliferation and viability and Ushio et al. (1991) also reported that the induction of mitochondrial activity of keratinocytes by geraniin. During development of human skin toward an intact barrier system, keratinocytes will undergo cellular proliferation followed by a switch to cellular differentiation to restore the breakage in the intact skin. Hence, the ability of PLE and geraniin to stimulate keratinocytes proliferation and increase cell viability of skin cells may suggest that they may promote the wound-healing process (Agyare et al., 2011). However, Lee et al. (2008) observed that geraniin can be proapoptotic at higher doses, which support the findings of our study.

## Conclusion

PLE and geraniin possess significant wound-healing activity. PLE and geraniin increased hydroxyproline production, collagen production, tensile strength of wound tissues, and TGF-β_1_ levels in wound bed and also exhibited good cytoprotective activity.

## Author Contributions

YB participated in the design of the study, carried out the laboratory analysis, and drafted the manuscript. CA conceived the concept, designed and coordinated the study, supervised the study, and revised the manuscript. GA participated in the laboratory analysis, analyzed the data obtained, and revised the manuscript. EA participated in the laboratory studies. NT participated in the design of the study and revised the manuscript. KD participated in the design of the study and revision of the manuscript. All authors read and approved the final manuscript.

## Conflict of Interest Statement

The authors declare that the research was conducted in the absence of any commercial or financial relationships that could be construed as a potential conflict of interest.
